# Supplementary material for: A shared decision-making intervention between health care professionals and individuals undergoing Pulmonary Rehabilitation: An iterative development process with qualitative methods
Source: PLoS One. 2024 Aug 19;19(8):e0307689. doi: 10.1371/journal.pone.0307689 (PMC11332919; doi:10.1371/journal.pone.0307689)
Supplement: S1 File — (DOCX) [file pone.0307689.s001.docx]

# Supplementary Materials

## S1: Collating data to inform the design of the PtDA

### Design steps 1 and 2a

To explore the conscious and verbalised factors which influence individuals’ decision-making for PR, qualitative semi-structured interviews were conducted with individuals living with COPD and PR healthcare professionals. A full description of the methods used are reported elsewhere (Barradell *et al.*, 2022).

### Design step 2b

To measure the presence of unconscious and non-verbalised factors which influence healthcare professionals interactions with individuals living with COPD, a computerised version of the Implicit Association Test (IAT; Greenwald, McGhee and Schwartz, 1998) was administered to healthcare professionals practicing within the UK. A full description of this step is reported elsewhere (Barradell *et al.,* 2023a).

### Design step 3

A systematic review was conducted to collate current evidence to inform PtDA development, content and look, delivery, and evaluation methods. The review methods are published elsewhere (Barradell *et al.,* 2023b).

### Design step 4

The steering group met to discuss and decide upon the attributes and data required for each option to display on the PtDA. Once these were decided, a narrative synthesis was conducted to collate the attributes and data required.

Data regarding the agreed attributes of each healthcare option were sourced via liaising with the host site’s PR service. Data regarding the agreed clinical outcomes of each healthcare option were sourced from real world data captured in the NACAP: PR workstream (NACAP, 2020), a Cochrane review exploring the evidence for telerehabilitation in people living with chronic respiratory disease (Cox *et al.*, 2021) and the official European Respiratory Society/American Thoracic Society technical standard on Cardiopulmonary Exercise Testing (Holland *et al.*, 2014). Data extracted for the clinical outcomes included the proportion of participants who met and or exceeded the cut off for the minimal clinically important difference (MCID). As the Cochrane review did not provide the MCID data required, individual study authors were contacted and asked to provide this.

## S2: Data to inform the design of the PtDA

### Design steps 1 and 2a

The results from the semi structured interviews with individuals and healthcare professionals on the barriers, facilitators and improvements for PR decision-making is reported elsewhere (Barradell *et al.*, 2022). Briefly, the results found that offering a choice of PR programmes was felt to support informed decision-making for PR. However, it was recognised that insufficient knowledge of the programmes would hinder this. Therefore, it was proposed that techniques to facilitate knowledge transfer and elicit individual-centred, meaningful discussions would be beneficial. The steering group agreed this provided justification to implement SDM using a tool to facilitate it (i.e. a PtDA).

### Design step 2b

The results from the IAT are reported elsewhere elsewhere (Barradell *et al.,* 2023a). In summary, the results indicated that healthcare professionals had implicit bias; they demonstrated a significant preference when associating exercise and pleasant attributes and smoking and unpleasant attributes. This suggested that they had negative attitudes towards individuals who smoke and positive attitudes towards those who exercise. This provided the steering group clarity that the SDM consultation should include a clear and robust structure to the SDM process which would enable healthcare professionals to support individual deliberation and decision-making without bias.

### Design step 3

The systematic review findings are reported elsewhere elsewhere (Barradell *et al.,* 2023b). To summarise, the review found evidence for the use of SDM interventions within chronic respiratory disease. Specifically, it led to the conclusion that a SDM intervention including a PtDA, healthcare professional training (i.e. decision coaching), and a consultation prompt would likely support individuals decision-making for PR. This, in combination with the results from Design step 2b and alpha testing (see findings below), provided the steering group with clarity on the need for supporting intervention components to facilitate PR SDM (i.e. decision coaching training and a consultation prompt).

### Design step 4

The steering group agreed that for each healthcare option, information regarding the content, frequency, location, support, feedback, advantages (e.g. likelihood of having a personalised COPD action plan, improvements in exercise capacity, quality of life), and disadvantages (e.g. accidents during exercise testing, adherence, likelihood of having an exacerbation of COPD) would support individuals’ understanding of the options and allow them to compare between each one systematically.

The COPD MCID data for each clinical outcome are tabulated below (Table 1). Whilst there were other measures of these outcomes, these were the most consistent measurement tools used across studies and therefore the ones chosen to illustrate the data on the PtDA.

Table 1: Current MCID data for selected clinical outcomes within the COPD population

| **Outcome** | **Measurement tool** | **MCID in COPD** |
| --- | --- | --- |
| Having a COPD action plan | Count | Not identified |
| Exercise capacity | 6-Minute Walk Test | 30m (Holland *et al.*, 2014) |
| Exercise capacity | Incremental Shuttle Walk Test | 35m (Evans and Singh, 2019) |
| Health-related quality of life | CRQ-Dyspnoea | 0.5 points (Jaeschke, Singer and Guyatt, 1989) |
| Health-related quality of life | mMRC Dyspnoea | 1 point (Mahler and Witek, 2005) |
| COPD exacerbations | Count | Not identified |
| Safety of exercise | Count | Not identified |
| Adherence to each option | Count | Not identified |

The synthesised data is displayed in Table 2. Two out of ten studies from the Cochrane review did not respond to the request for minimal clinically important difference data and therefore were not included.

Table 2: Synthesis of data for the PtDA

| **Outcome** | **Centre PR** | **Home PR - telephone** | **Home PR - online** | **Routine COPD care** |
| --- | --- | --- | --- | --- |
| Having a COPD action plan | 83 in 100 people (NACAP, 2020) | 83 in 100 people (NACAP, 2020) | 83 in 100 people (NACAP, 2020) | 24 in 100 people (Philip et al., 2019) |
| Having an MCID improvement in exercise capacity (6MWT) | 70 in 100 people (NACAP, 2020) | Between 32-40 in 100 people (Holland et al., 2016; Lahham et al., 2020) | Between 18-71 in 100 people (Tabak et al., 2014; Chaplin et al., 2017; Tsai et al., 2017; Knox et al., 2019; Hansen et al., 2020) | 0 in 100 people (Mccarthy et al., 2015) |
| Having an MCID improvement in health-related quality of life (CRQ-Dyspnoea) | 59 in 100 people (NACAP, 2020) | Between 35-90 in 100 people (Maltais et al., 2008; Holland et al., 2016; Lahham et al., 2020) | 18-60 in 100 people (Chaplin et al., 2017; Tsai et al., 2017; Knox et al., 2019) | 0 in 100 people (Mccarthy et al., 2015) |
| Having a COPD exacerbation | 7 in 100 people (Seymour et al., 2010) | 7 in 100 people (Seymour et al., 2010) | 7 in 100 people (Seymour et al., 2010) | 33 in 100 people (Seymour et al., 2010) |
| Having an adverse event during an exercise test | <1 in 100 people (Holland et al., 2014) | <1 in 100 people (Holland et al., 2014) | <1 in 100 people (Holland et al., 2014) | N/A |
| Adhering to the option | 42 in 100 people (NACAP, 2020) | Between 84-94 in 100 people (Maltais et al., 2008; Holland et al., 2016; Lahham et al., 2020) | Between 43-100 in 100 people (Tabak et al., 2014; Bourne et al., 2017; Chaplin et al., 2017; Tsai et al., 2017; Kwon et al., 2018; Knox et al., 2019; Hansen et al., 2020) | 70 in 100 people (Philip et al., 2019) |

## S3: Alpha testing tables of results

### Alpha testing with PR HCPs – proposed changes

| **Page Number** | **Section** | **Suggestion discussed with steering group** | **Component of the TFA** | **Action taken** |
| --- | --- | --- | --- | --- |
| 2 | What is COPD? | The term “blocking,” used to explain airway obstruction observed in COPD, was perceived negatively by the team. They suggested checking how this term is perceived by individuals as there may be a preferred or more accurate term.  Question posed to the group of individuals with COPD who reported this not to be a problem. | Affective attitude | No changes made. |
| 4 | What are my options for managing my COPD? | The team felt individuals would benefit from photographs to visually illustrate the differing COPD management options. | Intervention coherence | Not comparable for all options so may introduce bias. Steering group felt it was not appropriate to add this. |
| 6 | Step 1: Comparing the options | In the row entitled ‘How long does it last for?’ the team identified inaccurate terminology. They suggested changing the phrase “…at your own pace” to “…exercising daily at home.” | Intervention coherence | Changed. |
| 6 | Step 1: Comparing the options | In the row entitled ‘How is care delivered?’ the team identified inaccurate terminology. They suggested changing the phrase “In groups of other people living with COPD” to “In groups of other people with breathing difficulties.” | Intervention coherence | Changed. |
| 7 | Step 1: Comparing the options | In the row entitled ‘What are some disadvantages?’ they felt the word ‘complications’ was too vague and suggested using more specific terminology. | Intervention coherence | Changed to provide specific examples e.g., low oxygen levels, chest pain or a heart rate that is too fast. |
| 9 | Deciding between COPD management options | The team thought individuals would benefit from a clearer “blurb” in this section, particularly highlighting that individuals can choose multiple options here to discuss with their PR HCP and then refine their choice on page 11 (Step 4: What are the next steps?) | Intervention coherence | Added “you can choose more than one option” |
| 9 | Deciding between COPD management options | The team raised concerns about the time needed to go through the information with individuals and therefore requested they read up to page 9 before they meet with a PR HCP. | Affective attitude | Will encourage individuals to read and complete activities up to page 9 before they meet with their PR HCP. |
| Supplementary training component | - | The team felt they needed SDM training to upskill them and enable them opportunity to practice these skills prior to the feasibility study. | Self-efficacy | Opportunity to practice SDM skills added to the decision coaching training workshop. |
| Feasibility study | - | The team wanted study labels to be added to individuals’ healthcare records to show they required the intervention. | Intervention coherence | Agreed to record their enrolment into the feasibility study on their PR referral form |
| Feasibility study | - | The team wanted the ability to deliver the SDM intervention either face to face or by telephone as this would account for variations in service delivery following Covid-19. | Burden | Agreed that the intervention can be delivered by telephone or face to face. |
| Feasibility study | - | The team felt measuring intervention fidelity would be important to assess the efficacy of the intervention. | Perceived effectiveness | Agreed that an intervention fidelity measure will be included in the feasibility study. |
| Feasibility study | - | The team thought it would be important to undertake a qualitative appraisal of the intervention delivery from both theirs and a individuals’ perspective. | Perceived effectiveness | Agreed that a qualitative appraisal should be included in the feasibility study. |
| PtDA adoption (post-feasibility study) | - | The team felt that should the PtDA become part of routine clinical care it would be most appropriate for individuals to receive the PtDA upon referral to PR (i.e. to receive it in the waiting room or in the post with their appointment letter). | Intervention coherence | Agreed. But seek to explore this idea further in the feasibility study. |
| PtDA adoption (post-feasibility study) | - | The team felt should the PtDA become part of routine clinical care a systems-based approach should be adopted to ensure the intervention is adapted and provided to the entire PR referral population. | Ethicality | Agreed this would warrant additional investigation outside the scope of this study. |
| TFA: Theoretical Framework of Acceptability | | | | |

### Alpha testing with individuals - proposed changes

| **Page Number** | **Section** | **Suggestion discussed with steering group** | **TFA component** | **Actions taken** |
| --- | --- | --- | --- | --- |
| 1 | Logo | The group suggested adding some lungs to the outline of the person or a picture of lungs inside a circle of multiple pairs of arms. | Affective attitude | Logo ideas discussed to reach consensus. |
| 1 | Logo | The preferred option was a picture of lungs inside a circle of multiple pairs of arms to represent the different services available to individuals. | Affective attitude | Added. |
| 1 | Title | The group felt the wording “making decisions about your…” was better as it was more personal. | Affective attitude  Intervention coherence | Changed to include “your COPD” in the subtitle. |
| 2 | What is COPD? | The group thought we may need to include Covid-19 to this section. Otherwise the felt the language used was clear. | Intervention coherence | We thought this was not applicable because Covid-19 has its own rehabilitation programme. |
| 3 | Everyone’s experience of COPD is different | The group wanted a few more lines added for each question as they felt there was not enough space to respond to the questions. | Affective attitude  Intervention coherence | Added. |
| 3 | Everyone’s experience of COPD is different | The group wanted to add ratings to this section to enable individuals to look back on this later and see if there has been any change. | Intervention coherence | We felt this was too similar to the next section where it is unpicked in greater detail. The steering group agreed this section is more about starting to get people thinking about their COPD and its effect on their life and so does not require a rating scale. |
| 4 | What are my options for managing my COPD? | The group felt there was a difference between community and hospital-based PR in the sense that community-PR is likely to include locals to you. They suggested this may mean the ability to create more friendships. They therefore felt including the term ‘local’ would support individuals’ reflections on this. | Intervention coherence | Have added the word ‘local’ before each mention of community venue to highlight difference. |
| 4 | Throughout PtDA | The group felt the references would be best written in brackets following research data as follows: (Author, Year). | Intervention coherence | Changed to Harvard referencing format. |
| 4 | What are my options for managing my COPD? | The group would prefer the terminology Pulmonary Rehabilitation and then PR afterwards. And GP Practice. | Intervention coherence | Changed. |
| 5 | What is important to me in my daily life? | The group suggested adding a rating scale of 1-5 to show how much influence each factor has. | Intervention coherence | Added. |
| 5 | What is important to me in my daily life? | The group suggested adding support from external groups (e.g. social/sport groups…) as this could highlight interest in other options and provide a prompt for PR team to give list of clubs. | Affective attitude | The steering group felt this is covered under support from others with COPD and so no change made. |
| 5 | What is important to me in my daily life? | The group recognised the wait time to start PR is different between each option (e.g. home-based PR may start quicker than centre-PR as depends on waiting list) and felt this should be highlighted. | Intervention coherence | Added a ‘when can I start it?’ row. |
| 6-7 | Step 1: Comparing the options | The group would prefer to combine the information here and have it presented in landscape as it “would be easier to read” and “easier to compare.” They also felt it important to update this section when new information comes to light. | Intervention coherence  Perceived effectiveness | Changed page orientation to landscape. |
| 7 | Step 1: Comparing the options | The group felt in the row titled ‘What are the disadvantages?’ we should consider changing the word “complications” to something more specific. | Intervention coherence | Provided examples of possible complications for clarity. |
| 7 | Step 1: Comparing the options | One individual stated “I have re-read the document and I understand its use.  The main point I would make is regarding page 7. It is a mass of information which may well be off-putting.  The average person may not be aware of the use of academic references which could be a little confusing.  But I also wonder whether the use of the statistics will put people off from reading it thoroughly.  It may be more straightforward to list each option in turn and state the advantages and disadvantages in more general terms. | Intervention coherence | 1 person said this, 4 others felt it was okay to have the information in tables as it helped with comparing the options against each other so the steering group agreed to leave the data presentation as it is. |
| 7 | Step 2: My views about the options | The group suggested adding a rating scale to this section. | Intervention coherence | Rating scale added. |
| 8 | Step 1: Comparing the options | They felt there wasn’t much information in the tables and so it would be worth adding more information about travelling to venues, location of venues, type of transport options available. | Intervention coherence | Added another column called ‘What do I need to do to access it?’ |
| 9 | Deciding between COPD management options | The group recommended changing the question to “Are there any questions you want to ask a PR HCP at your appointment?” | Intervention coherence | Added. |
| 10 | Step 3: Preparing to make plans | The group felt this section was easy to understand apart from last question. They didn’t understand the word “balanced” and so felt this was worth removing or re-phrasing. | Intervention coherence | Changed to how “equally” the information is presented. |
| 11 | Step 4: What are the next steps? | The group felt we could add a question to about wanting more information about additional fitness/social groups for COPD. | Affective attitude | The steering group felt this was not the focus of this PtDA so have left out for now. |
| Supplementary overview | Throughout | The group identified that it says “check” next to each rating scale and thought “tick” would be more appropriate. | Intervention coherence | Have changed to “tick” throughout. |
| Supplementary training | - | The group felt training the PR team was a good idea and should be standardised so the team deliver the intervention in the same way (e.g. provide a script, protocol). | Self-efficacy  Ethicality | In the PR decision coaching training we added an activity for PR HCPs to write their own SDM consultation prompt. |
| TFA: Theoretical Framework of Acceptability | | | | |

## S4: Finalised PtDA (preview; pages 1, 7 and 13 of 17 page booklet. Subject to copyright version 6)

**Making Decisions about Chronic Obstructive Pulmonary Disease (COPD)**


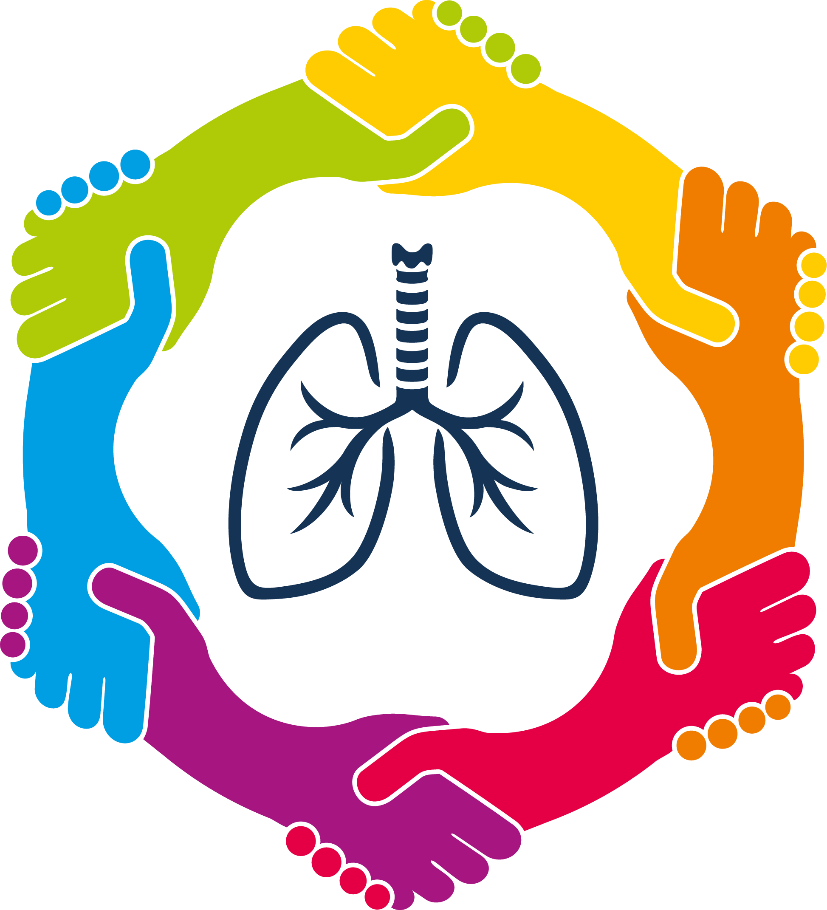


**A leaflet to help you make decisions about how best to manage your COPD, with or without Pulmonary Rehabilitation**


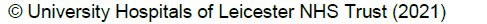


##

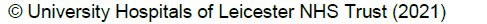


##

##
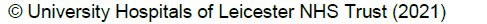


## S5: User-centered design process

Table 3: Adherence to the DEVELOPTOOLS user-centred design checklist

| **Checklist item** | **Adherence** |
| --- | --- |
| *Factor: Preprototype involvement*  1. Were potential users involved in any steps to help understand users (e.g., who they are, in what context might they use the tool) and their needs? | Yes – in depth interviews were conducted with patients and healthcare professionals to explore the decision-making needs of individuals considering PR (see Design steps 1 and 2a). |
| 2. Were potential users involved in any steps of designing, developing, and/or refining a prototype? | Yes – a patient advisor from the steering group reviewed a draft design of the patient decision prior to its development. They also contributed to the design, development and refinement of the PtDA. |
| *Factor: Iterative responsiveness*  3. Were potential users involved in any steps intended to evaluate prototypes of the tool or a final version of the tool? | Yes – comprehensibility and usability testing was conducted with 4 patients. They reviewed 2 iterations of the prototype (see Alpha testing. |
| 4. Were potential users asked their opinions of prototypes of the tool or a final version of the tool in any way? | Yes – 4 patients provided their overall opinions on 2 iterations of the prototype (see Alpha testing). |
| 5. Were potential users observed using the tool in any way? | No - no formal observation was conducted, however, patients pretended to use it whilst reviewing the prototype (see Alpha testing). |
| 6. Did the development process have 3 or more iterative cycles? | Yes – the finalised PtDA was version 10 (see Figure 2 and Prototype development). |
| 7. Were changes between iterative cycles explicitly reported in any way? | Yes (see Figure 2 and Prototype development). |
| *Factor: Other expert involvement*  8. Were health professionals asked their opinion of the tool at any point? | Yes – acceptability and usability testing was conducted with 8 PR specialists (see Alpha testing). |
| 9. Were health professionals consulted before a first prototype was developed? | No - no formal consultation was conducted with healthcare professionals, however, brief conversations about the nature of the prototype were conducted. Formal consultations with healthcare professionals began once the prototype was developed. |
| 10. Were health professionals consulted between initial and final prototypes? | Yes - acceptability and usability testing was conducted with 8 PR specialists (see Alpha testing). |
| 11. Was an expert panel involved? | Yes – the tool was development in collaboration with a multi-disciplinary steering committee (see The steering group). Additional support was provided by a graphic designer who created the PtDA logo and advised on the graphic layout and structure of the PtDA. |

References

Barradell AC, Bourne C, Alkhathlan B, Larkin M, Singh SJ (2022). A qualitative assessment of the pulmonary rehabilitation decision-making needs of patients living with COPD. *NPJ Prim Care Respir Med*;32(1):23. doi: 10.1038/s41533-022-00285-9. PMID: 35768417; PMCID: PMC9243001.

Barradell AC, Robertson N, Houchen-Wolloff L, Singh SJ (2023a). Exploring the presence of implicit bias amongst healthcare professionals who refer individuals living with COPD to Pulmonary Rehabilitation with a specific focus upon smoking and exercise. *International Journal of Chronic Obstructive Pulmonary Disease* (accepted, awaiting publication)

Barradell AC, Gerlis C, Houchen-Wolloff L, Bekker HL, Robertson N, Singh SJ (2023b). Systematic review of shared decision-making interventions for people living with chronic respiratory diseases. *BMJ Open*: 2;13(5):e069461. doi: 10.1136/bmjopen-2022-069461. PMID: 37130669; PMCID: PMC10163462

Bourne, S., Devos, R., North, M., et al. (2017) ‘Online versus face-to-face pulmonary rehabilitation for patients with chronic obstructive pulmonary disease: randomised controlled trial’, *BMJ Open*;, 7(7), p. e014580. doi: 10.1136/BMJOPEN-2016-014580.

Chaplin, E., Hewitt, S., Apps, L., et al. (2017) ‘Interactive web-based pulmonary rehabilitation programme: a randomised controlled feasibility trial.’, *BMJ open*; 7(3), p. e013682. doi: 10.1136/bmjopen-2016-013682.

Cox, N. S., Dal Corso, S., Hansen, H., et al. (2021) ‘Telerehabilitation for chronic respiratory disease’, *Cochrane Database of Systematic Reviews. John Wiley and Sons Ltd*. doi: 10.1002/14651858.CD013040.pub2.

Evans, R. A. and Singh, S. J. (2019) ‘Minimum important difference of the incremental shuttle walk test distance in patients with COPD’, Thorax. *BMJ Publishing Group*, 74(10). doi: 10.1136/thoraxjnl-2018-212725.

Greenwald, A. G., McGhee, D. E. and Schwartz, J. L. K. (1998) ‘Measuring individual differences in implicit cognition: The implicit association test’, *Journal of Personality and Social Psychology. American Psychological Association Inc*., 74(6), pp. 1464–1480. doi: 10.1037/0022-3514.74.6.1464.

Hansen, H., Bieler, T., Beyer, N., et al. (2020) ‘Supervised pulmonary tele-rehabilitation versus pulmonary rehabilitation in severe COPD: A randomised multicentre trial’, *Thorax*; 75(5), pp. 413–421. doi: 10.1136/thoraxjnl-2019-214246.

Holland, A. E., Spruit, M. A., Troosters, T. et al. (2014) ‘An official European respiratory society/American thoracic society technical standard: Field walking tests in chronic respiratory disease’, *European Respiratory Journal;*  44(6), pp. 1428–1446. doi: 10.1183/09031936.00150314

Holland, A. E., Mahal, A., Hill, C. J., et al. (2016) ‘Home-based rehabilitation for COPD using minimal resources: a randomised, controlled equivalence trial’. Thorax; 72, 507-65, doi: 10.1136/thoraxjnl-2016-208514.

Jaeschke, R., Singer, J. and Guyatt, G. H. (1989) ‘Measurement of health status. Ascertaining the minimal clinically important difference’, *Controlled Clinical Trials;* 10(4), pp. 407–415. doi: 10.1016/0197-2456(89)90005-6

Knox, L., Dunning, M., Davies, C. A., et al. (2019) ‘Safety, feasibility, and effectiveness of virtual pulmonary rehabilitation in the real world’, *International Journal of COPD;* 14, pp. 775–780. doi: 10.2147/COPD.S193827.

Kwon, H., Lee, S., Jung, E. J., et al. (2018) ‘An mHealth management platform for patients with chronic obstructive pulmonary disease (Efil breath): Randomized controlled trial’, *JMIR mHealth and uHealth;*  6(8). doi: 10.2196/10502.

Lahham, A., McDonald, C. F., Moore, R., et al. (2020) ‘The impact of home-based pulmonary rehabilitation on people with mild chronic obstructive pulmonary disease: A randomised controlled trial’, *Clinical Respiratory Journal;* 14(4), pp. 335–344. doi: 10.1111/crj.13138.

Mahler, D. A. and Witek, T. J. (2005) ‘The MCID of the Transition Dyspnea Index is a total score of one unit’, in COPD: *Journal of Chronic Obstructive Pulmonary Disease;* pp. 99–103. doi: 10.1081/COPD-200050666.

Maltais, F., Bourbeau, J., Shapiro, S., et al. (2008) ‘Effects of home-based pulmonary rehabilitation in patients with chronic obstructive pulmonary disease: A randomized trial’, *Annals of Internal Medicine;* 149(12), pp. 869–878. doi: 10.7326/0003-4819-149-12-200812160-00006

Mccarthy, B., Casey, D., Devane, D., et al. (2015) ‘Pulmonary rehabilitation for chronic obstructive pulmonary disease’, *Cochrane Database of Systematic Reviews.* doi: 10.1002/14651858.CD003793.pub3.

NACAP (2020) National Asthma and Chronic Obstructive Pulmonary Disease Audit Programme (NACAP). Pulmonary rehabilitation clinical audit 2019. Clinical audit of pulmonary rehabilitation services in England, Scotland and Wales. Patients assessed between 1 March and 31 M. London.

Philip, K., Gaduzo, S., Rogers, J., et al. (2019) ‘Patient experience of COPD care: outcomes from the British Lung Foundation Patient Passport’, *BMJ Open Respiratory Research*; 6(1), p. e000478. doi: 10.1136/bmjresp-2019-000478.

Seymour, J. M., Moore, L., Jolley, C. J., et al. (2010) ‘Outpatient pulmonary rehabilitation following acute exacerbations of COPD’, *Thorax,* 65(5), pp. 423–428. doi: 10.1136/thx.2009.124164.

Tabak, M., Brusse-Keizer, M., van der Valk, P., et al. (2014) ‘A telehealth program for self-management of COPD exacerbations and promotion of an active lifestyle: A pilot randomized controlled trial’, *International Journal of COPD*; 9, pp. 935–944. doi: 10.2147/COPD.S60179.

Tsai, L. L. Y., McNamara, R. J., Moddel, C., et al. (2017) ‘Home-based telerehabilitation via real-time videoconferencing improves endurance exercise capacity in patients with COPD: The randomized controlled TeleR Study’, *Respirology;* 22(4), pp. 699–707. doi: 10.1111/resp.12966
