# Supplementary material for: A shared decision-making intervention between health care professionals and individuals undergoing Pulmonary Rehabilitation: An iterative development process with qualitative methods
Source: PLoS One. 2024 Aug 19;19(8):e0307689. doi: 10.1371/journal.pone.0307689 (PMC11332919; doi:10.1371/journal.pone.0307689)
Supplement: Attachment — Submitted filename: PLOS ONE final reviewer letter.docx [file pone.0307689.s002.docx]

03.04.2024

Dear Tamer I. Abo Elyazed, Ph.d

**RE: PONE-D-23-33990**

**A Shared Decision-Making intervention for individuals living with COPD who are making Pulmonary Rehabilitation treatment options with respiratory and physiotherapy healthcare professionals: An iterative development process with qualitative methods**

**PLOS ONE**

Thank you for the opportunity to revise our manuscript. We have responded to the reviewer comments point by point below and have uploaded a tracked changes and final copy of the revised manuscript.

- **Additional Editor Comments (if provided):** The title of the manuscript needs to be re structured to clarify the goal of the study in more simple abbreviated words; e.g I suggest [A shared decision making between health care professionals and individuals undergoing Pulmonary rehabilitation : An iterative development process with qualitative methods]. Thank you we have amended to: ‘A shared decision- making intervention between health care professionals and individuals undergoing Pulmonary rehabilitation: An iterative development process with qualitative methods’.
- **Reviewer 1:** **Limited Uptake Data:** The intervention was completed two years ago, yet there is a lack of data on the uptake of the three interventions. While it is acknowledged that the authors might intend to publish this in a separate article, the absence of uptake data in the current study limits a comprehensive assessment of the intervention's real-world impact. The focus of this paper is the development of the intervention and therefore precedes any examination of its feasibility/ uptake. The protocol paper (reference 44) is included in the methods to show where this development works fits into the full body of work.
- **Reviewer 1: Small Alpha Test Sample:** The alpha-testing phase involved a small number of participants, with 7 (8) healthcare professionals and 3 (4) patients. The limited size of the alpha test group raises questions about the generalizability and robustness of the intervention, particularly in capturing a diverse range of perspectives. Added to limitations (page 17): ‘however, we recognise that we had a small number of individuals who contributed. It may be that we did not capture the diversity of opinions of all individuals living with COPD, however we did obtain rich, in depth data from this qualitative work’
- **Reviewer 1: Absence of Beta Testing Data:** The study lacks beta testing data conducted in real-life conditions (field tests) about feasibility. In the PtDA development model utilized by the authors, based on Coulter et al.'s 2013 article, Coulter et al. have outlined both alpha testing, focusing on acceptability and usability, and beta testing, which includes real-world field testing for feasibility. However, the presentation lacks data or reflections on why only alpha test findings are presented, and whether the PtDA development process concluded after alpha testing without progressing to beta testing. It is possible that the authors chose to deviate from the model, adopted a different approach, or pursued an alternative path, but there is no available data or reflections on this matter. Therefore it appears, that the PtDAs appear to lack comprehensive field testing with patients or evaluation by clinicians not involved in the development process, undermining the understanding of its feasibility in practical settings. Beta testing is in the feasibility study- to be published later. As above, the focus of this paper is the development of the intervention and therefore precedes any examination of its feasibility/ uptake. The protocol paper (reference 44) is included in the methods to show where this development works fits into the full body of work.
- **Reviewer 1: Timeline Discrepancy:** Reference is made to the use of the user-centered design checklist (reference 41) at the early stages of the study, during the initial phase of outlining the scope of the intervention. However, the timeline raises concerns, as reference 41 was published in October 2021, several months after the project's initiation in March 2021 (although reference 41 was online mid-June this is still several months after the start of this project)? So was this incorporated semi-retrospective? An earlier version of the guidance was used to inform the study design – originally from two sources:

First the following reference on user centred design checklist (2018):

file:///C:/Users/epihlb/OneDrive%20-%20University%20of%20Leeds/Hilary/collaborators/Holly%20Witteman/UCD_11_2018.pdf

Second the developing patient decision aids guidance 2013:

<https://bmcmedinformdecismak.biomedcentral.com/articles/10.1186/1472-6947-13-S2-S2>

The publication in 2021 brings both these patient decision aid guidance tools together. If needed, both of the earlier references can be added.

- **Reviewer 1: Sequential Figure Reference:** On page 7 of the manuscript, Figure 2 is referenced before Figure 1. Typically, figures and tables should be referenced consecutively in a scientific article. Maybe this edit will be addressed during the proofreading process to ensure consistency and compliance with common scientific standards. We have changed the order of figures in the text (page 8).
- **Reviewer 1: Imbalance in Steering Group Composition:** The steering group comprises 5 specialists/academics/experts and only 1 patient. This composition may not sufficiently address the inherent power imbalance, potentially limiting the representation of the patient's voice in decision-making. This should be reflected on and argued why only one patient representative was included. Whilst we acknowledge your point, we would argue that we have 1-2 people per stakeholder group: individual living with COPD (n=1), clinical academic specialist in pulmonary and cardiac rehabilitation (n=2), specialist in clinical and health psychology (n=1), trainee health psychologist (n=1), and academic specialist in medical and SDM (n=1) so the group was balanced. However we have added to limitations (page 17): ‘Furthermore, the steering group comprised of 5 academics/experts and only 1 patient. This composition may not sufficiently represent the patient's voice in decision-making.’
- **Reviewer 1: Unclear Scope of Consultation Prompt:** The manuscript lacks information about the scope, appearance, and specific content of the consultation prompt. In my view, this part of the intervention is too implicit, and it is unclear to readers what specifically it entails. A more detailed explanation is needed to understand the intervention's purpose and what exactly this is in the context of the study. Added more detail to the results page 15: ‘The finalised SDM intervention consisted of three distinct components: decision coaching training for PR HCPs, the PtDA, and a SDM consultation which was guided by a consultation prompt. This was individually developed by the PR HCPs during the decision coaching training (Figure 4) and consisted of individualised prompts and conversation starters to guide the SDM consultation.’
- **Reviewer 1: Limited Display of PtDA:** Only the front page of the PtDA is presented in S4: “Finalised PtDA (preview; version 6). “To provide a comprehensive understanding, the entire PtDA should be showcased, as the front page alone does not convey sufficient information about its contents. A couple of extra pages of the PtDA have been added to the supplement, subject to copyright.

**Reviewer 2: Abstract**

**•**May have missed this, but a ‘useable leaflet’ only appears to be referenced in the abstract, with no mention of what this looks like elsewhere within the paper. It would be good to expand. Used the term ‘format’ in the abstract instead to avoid confusion.

**Reviewer 2: Abstract**

**•**The authors provide a comparison of an online PR programme vs. traditional programme, but more information about what a traditional programme looks like would be beneficial. Readers of PLOS ONE typically come from different backgrounds and may not be familiar. Would be good to provide more context to the area. We have expanded the explanation of PR in the introduction (page 4): ‘Traditionally PR is delivered face to face in a hospital or community setting over 6-8 weeks, with at least 2 supervised sessions per week. The programme consists of both resistance and endurance exercise, in addition to self-management education’. Also by adding extra pages of the PtDA to the supplement, this provides extra description of the traditional, centre-based PR.

**•**Similarly, commonly cited barriers to the uptake of traditional PR (e.g. organisational constraints) are mentioned, but an expansion and explanation of these barriers would be good. Given examples on page 5: ‘Commonly cited barriers included organisational constraints [e.g. limited timings/ locations for PR (20)]’.

**Reviewer 2: Methods**

**•**Step 4 describes an exercise used to support individuals identifying core values and beliefs, but a description of what this entailed would be beneficial. Added to page 9: ‘This involved a process of identifying what matters to an individual relevant to their given health decision (i.e. PR decision). Patients used a section of the PtDA to complete ‘What is important to me in my daily life?’ and were asked to rate on a scale of 1-5 from ‘not important’ to ‘extremely important’ items such as ‘daily activities in the home’, ‘work’, spending time with family’, ‘understanding my COPD’ etc’.

**•**A ‘think-aloud approach’ is described in the Alpha testing section. I wonder if the authors have any quotes they could present to support their findings**.** There are several quotes in the supplementary material, table S3. We have added a quote to support this on page 10: ‘E.g. on the topic of ‘Deciding between COPD management options’, the patient group recommended changing the question to “Are there any questions you want to ask a PR HCP at your appointment?”’

**Reviewer 2: Results**

**•**It is interesting that the HCPs seemed to identify burden as one area that required attention, but the PPI group did not. I wonder if the authors had any thoughts as to why this was the case? Would be interesting to discuss. Add to discussion, page 17: ‘Interestingly, the time burden was not an area that patients identified as a barrier. It may be that the patients would relish the additional time to discuss PR option with their healthcare provider, whereas clinicians were concerned about including the SDM intervention within an already time-pressured assessment period.’

**Reviewer 2: Discussion**

**•**In one area, the authors talk about limited research on the barriers of SDM, with one being insufficient knowledge of SDM amongst HCPs. The authors could explore whether gaining more knowledge always leads to taking action. In the field of behaviour change, it has been observed that knowing what to do does not always result in actual behaviour change. It would be valuable for the authors to contemplate this aspect in the context of potential future research. Added this point to the discussion, page 17: ‘That said gaining more knowledge on SDM may not necessarily lead to behaviour change, therefore the link between increased knowledge and taking action should be addressed/ measured in future research’.

We look forward to hearing from you in due course.

Yours sincerely,

Linzy Houchen-Wolloff PhD

Corresponding author on behalf of all co-authors.
