# Supplementary material for: A shared decision-making intervention between health care professionals and individuals undergoing Pulmonary Rehabilitation: An iterative development process with qualitative methods
Source: PLoS One. 2024 Aug 19;19(8):e0307689. doi: 10.1371/journal.pone.0307689 (PMC11332919; doi:10.1371/journal.pone.0307689)
Supplement: Attachment — Submitted filename: PLOS ONE revision letter2.docx [file pone.0307689.s003.docx]

08.07.24

Dear Tamer I. Abo Elyazed, Ph.d

**RE: PONE-D-23-33990 Revision 2**

**A Shared Decision-Making intervention for individuals living with COPD who are making Pulmonary Rehabilitation treatment options with respiratory and physiotherapy healthcare professionals: An iterative development process with qualitative methods**

**PLOS ONE**

Thank you for the opportunity to revise our manuscript. We have responded to the remianing reviewer comment below and have uploaded a tracked changes and final copy of the revised manuscript.

**Reviewer #1: Thank you for the sufficient response to the raised criticism. I believe the article is ready for publication. However, I don't think it is clear to the reader when reading the figure legends for supplementary figure 4 that it is only a small excerpt of figure 4 that is presented in the supplementary material. I find it unfortunate that they do not display the entire PtDA in supplementary figure 4. However, if the authors are concerned due to copyright issues, it must be taken into account. Nevertheless, they should explain to the reader that only a small excerpt is shown in the supplementary material, for example, that it is page 1, x, and x out of a total of xx pages of the paper-based (?) PtDA.**

In the manuscript- it is made clear that only a small excerpt is shown.

In the supplement- the following text has been added: ‘S4: Finalised PtDA (preview; pages 1, 7 and 13 of 17 page booklet. Subject to copyright version 6)’

We look forward to hearing from you in due course.

Yours sincerely,

Linzy Houchen-Wolloff PhD

Corresponding author on behalf of all co-authors.
